# Gaps Between Self-Reported and Documented Use of a Health Care Service Portal Among Older Adults: Cross-Sectional Survey and Retrospective Electronic Data Study

**DOI:** 10.2196/93057

**Published:** 2026-10-13

**Authors:** Efrat Neter, Diane Levin-Zamir, Ester Eliyahu, Peter W S Chang, Tuyen V Duong, Orna Baron-Epel

**Affiliations:** 1 Behavioral Sciences Department, Ruppin Academic Center, 3 Bait, Emeq Hefer, 4025000, Israel, 972 0546462677, 972 98983043; 2 School of Public Health, Faculty of Social Welfare and Health Sciences, University of Haifa, Haifa, Israel; 3 Department of Health Education and Promotion, Clalit Health Services, Tel-Aviv, Israel; 4 Chang Bing Show Chwan Memorial Hospital, Lukang, Taiwan; 5 School of Nutrition and Health Sciences, Taipei Medical University, Taipei, Taiwan

**Keywords:** digital health literacy, ethnicity, portal use, online portal, older adults

## Abstract

**Background:**

Health care systems increasingly offer services to citizens through online portals, which have been found to be associated with patient satisfaction and better management of health concerns. Few studies have compared self-reported use with objectively documented portal use, especially among older adults.

**Objective:**

This study aimed to examine differences between documented and self-reported health portal use, and their associations with background variables and digital health literacy (DHL) among older adults.

**Methods:**

A cross-sectional telephone survey was conducted among 600 participants aged 60 years and older, who were sampled from a registry of members of a national health maintenance organization (HMO). The participants reported their use of the HMO’s portal, their DHL, and demographic and background variables. Participants’ use of the portal was retrospectively retrieved from the HMO’s administrative database.

**Results:**

A large gap between self-reported use and documented use of the health portal was found: for example, while only 8.1% (46/566) reported not using the portal, the HMO’s records indicated that 62% (372/600) did not make use of the portal. However, a positive correlation was found between self-reported use and documented use (*r*=0.22; *P*<.001). Significant positive associations were found between administrative and physician-related online services, both reported use (*r*=0.41; *P*<.001) and documented use (*r*=0.56; *P*<.001). A multivariate analysis, where documented use was the dependent variable, explained 12.5% of the variance. Experience with computers (β=0.13; *P*=.02) and ethnicity (β=−0.22; *P*<.001) were significantly associated with documented use. DHL, though significantly associated with documented use at the bivariate level (*r*=0.09; *P=*.04), was not significantly associated with documented use in this analysis (β=0.02; *P*=.97).

**Conclusions:**

The findings indicate that users tend to overestimate their health portal use. Interpretations vary from measurement issues, such as social desirability bias regarding digital functioning or the perception that assisted use also constitutes usage, to signaling their identity as engaged digital health consumers. Future studies on digital health use should also include measures of electronically documented use and not only self-reported use.

## Introduction

Digital transformation in health care, and its potential for improving patient outcomes, operational efficiency, and care quality, is fundamentally contingent on users’ active engagement and their digital competencies. Consistent evidence demonstrates that the success of digital health technologies critically depends on users’ willingness to participate in and effectively use these systems [1,2]. Individuals need sufficient digital health literacy (DHL) to access, understand, appraise, and apply health information from electronic sources to realize the benefits of digital health solutions [3]. The evidence indicates that without adequate user engagement and digital skill development, digital health technologies often produce limited clinical impact [4].

A patient health portal is a digital health application, defined as a secure, web-based platform through which patients can access and manage their personal health services, including, for example, communication with health care providers (telehealth), medical records, laboratory results, appointment scheduling, prescription requests, and referrals. The Danish national health portal, sundhed.dk, exemplifies the use of these platforms for understanding laboratory results and treatment plans, with the authors reporting on actual patient use [5]. Systematic reviews from recent years on the use of health portals and their outcomes [6-8] surveyed the different metrics, reported on positive associations with health processes (monitoring, interactions with health care providers, and health behaviors), and noted that patient portals are increasingly integral to health systems, offering functionalities that support patient engagement. Still, the quality of evidence on the effects of service use (eg, health care workers’ workload, emergency department use, and hospitalization) and clinical outcomes was rated as low to moderate.

When discussing patient portal use, it is important to distinguish between self-reported use and documented use, and the current study focuses on the gap between the two. Reported use refers to data gathered directly from patients through surveys, interviews, or questionnaires, where they self-report their portal activities, perceptions, attitudes, or intentions [9]. Numerous studies have focused on reported use of portals in conjunction with self-reported eHealth literacy [10,11]. Documented use, on the other hand, refers to objective data tracked directly from patient portal systems, such as login frequency, session duration, specific functionalities accessed (eg, viewing test results and sending messages), digital health outcomes (health behaviors and clinical outcomes), and health care use patterns (interactions with health care providers) [12].

Few studies have reported on both documented and reported use. For example, Woods et al [10] examined patient factors associated with documented use among patients (n=270) who registered with a portal and self-identified as users and reported the percentage of documented users among those reporting use. It should be noted that studies in a related field of DHL, which assessed both reported and actual literacy levels, found a low to medium association between the two [11-13]. A comparison of self-reported use to documented use may serve as a form of validation of self-reported use. The question is, to what degree does self-reported use of portal services represent actual or documented use, as measured by computerized measures of use?

A related research effort that focused on the factors associated with adoption and engagement by patients with their portals categorized these factors into demographic (age, gender, and race/ethnicity), socioeconomic (education, income, insurance, and geography), health-related (chronic conditions/health status and patient activation), and technology-related characteristics (health literacy, digital access/skills, and DHL). There is considerable heterogeneity in the findings, and a systematic review [7], which analyzed 32 studies on this subject, identified only women’s gender and age (older, though depending on the population studied) as consistently associated with portal use. Another umbrella review [6] did not find strong evidence of an association between patients’ characteristics and portal use, and assessed the quality of the evidence (regarding private insurance, higher illness burden, race [White], age, and income) as moderate.

Studies that examined both self-reported and documented use of health portals are scarce and are often based on small samples, presenting a research gap. The objective of the present study was to address this gap by examining both types of use in a medium-sized sample, assessing the association between the two, and examining patient characteristics associated with documented and self-reported use. We focused on the population of older adults (aged >60 years), who represent a growing demographic that often faces challenges in managing complex and multiple chronic conditions, leading to a high demand for health care services. Due to their increased health care needs and frequent interactions with the health care system, they are also considered among the populations that may benefit significantly from patient portals for improved access to care, self-management, communication, and health information [14]. In addition to demographic and socioeconomic characteristics, we also included the often-studied skill of DHL, which has been found to be associated with engagement and use [15].

We hypothesized the following:

- H1: Reported use of the health portal will be higher than documented use, and the two use measures will be positively associated with each other to a weak-to-medium degree.
- H2: Patient characteristics will be associated with both reported and documented use. Based on the cited reviews, we hypothesize that, in the restricted age group of those older than 60 years, age will be negatively associated with use measures, while women will have higher documented and reported use than men.
- H3: DHL will be positively associated with both reported and documented use.

In addition, we explored the following research question (RQ): Are there associations between portal-documented use and socioeconomic status, self-rated health, and prior use of the internet and computers?

## Methods

### Sample

The eligibility criteria for the study included being a member of “Clalit” Health Services (CHS) in Israel, being aged 60 years or older, and being fluent in Hebrew, Arabic, or Russian.

CHS is Israel’s largest health maintenance organization (HMO), serving more than half of the country’s population. It is a nonprofit organization, sanctioned by Israel’s National Health Insurance Law of 1994, operating a comprehensive network of community and hospital services throughout the country, and, over the past several decades, has maintained electronic health records that support clinical care, quality improvement, and both clinical and population-based research [16,17]. CHS has a digital portal that includes a large, diverse range of services and can be accessed from any type of digital device (mobile phone, personal computer, laptop, or tablet). Access to the portal—for both patients and health care providers—is availed by using the patient’s personal identification number.

A random list of potential participants (names and phone contact details) was generated by CHS, assigned a serial number, anonymized, and securely transferred to a survey company. The target number of 600 (actually, N=601) was reached after phone contact was initiated with 5688 individuals. The overall response rate was 20.4%, with a sampling error of 2.04%, discounting phone numbers with no response after at least 3 attempts. The phone interviews were conducted in Hebrew, Arabic, or Russian, according to the preference of the interviewee. There was no requirement for prior experience using the portal, as all CHS members are entitled to access the digital resources.

### Design and Procedure

The telephone survey’s design was cross-sectional, while the data on documented portal use were retrospective for the year 2024. The outcome (dependent) variables were the documented and reported use of the health portal. The independent variables were demographics, DHL, and use of digital resources and devices. The survey was conducted from June to September 2024. Following the telephone survey, information on the documented use of the CHS portal was extracted from the national CHS information system on “no-queue” services, matched to each individual who responded to the survey, and anonymized for analysis.

### Measures

#### Documented Portal Use

CHS collects information on the use of its website/mobile application through which digital health services are provided. Service use is recorded upon completion of each service transaction. Portal use pertains to information regarding the types of services. We grouped services (with equal weights) into medical/health, administrative, and overall services. The medical/health services include medical authorizations/certificates (eg, sick leave) and clinical summaries; results of tests/procedures and referrals; and requests for prescriptions (eg, refills of routinely taken medications). The administrative services include consultations with clinic administrators/service staff, authorizations for funding of medical procedures implemented outside the CHS system, and payment refunds.

#### Reported Portal Use

Respondents were asked about the level of use of the above-mentioned services on the CHS portal during the past 6 months (Multimedia Appendix 1). The list of services was read out to the respondents, who estimated the number of times they accessed the portal. The 6-month time frame was chosen as this is a time frame widely used in health care use, found to produce more accurate reporting (than 12 mo) and lower the chances of recall bias [18]. Responses were recorded on a 5-point scale (not at all, less than once in this time period, 1‐2 times, 4‐6 times, and more than once in a given month). We included the response option “less than once in this time period” to convey the possibility that respondents have used the activity sometime in the past, but not in the time period asked about, as distinct from “not at all.” Three indices of reported portal use were constructed by averaging across service items: reported medical/health-related use (Cronbach internal reliability was α=0.71), reported administrative use (Cronbach internal reliability was α=0.59), and overall reported use.

#### DHL

DHL was measured using the HLS_19_-DIGI instrument [19] of the World Health Organization Action Network on Measuring Population and Organizational Health Literacy, which comprises 8 items assessing the difficulty or ease of the following skills: accessing, understanding, appraising, and applying online health information. The internal reliability (Cronbach α) was 0.89 in the current study.

As a correlate of DHL, an additional module (HLS_19_-DIGI-DD), comprising 6 items, assessed the frequency of using digital health resources. Participants reported the frequency (ranging from 1=“Not relevant or less than once per week” to 5=“More than once per day”) of using the following resources: websites, social media, digital devices, mobile health apps, digital communication with health services, and “other digital resources.”

#### Demographic Characteristics

Participants provided information on gender, age, country of birth, ethnicity/religion (Jewish, Muslim, Christian, Druze, and other; grouped such that Muslims, Christians, and Druze were classified as Arab, and Jews and others were classified as Jewish), religiosity, education (grouped into less than high school, completed high school, and completed more than high school), perceived social status (the MacArthur Scale [20]), difficulty covering the cost of medications, employment status, marital status, household size, and self-rated health [21].

### Ethical Considerations

This study was approved by the institutional ethics review boards of both the University of Haifa (#298/25) and CHS (#0101‐23-COM2). All respondents expressed explicit consent before their participation, and their consent was orally recorded; they were notified that any publication of the study would contain no identifying information. All data were anonymized for data analysis purposes. No compensation was offered or given to participants.

### Statistical Analysis

Participants’ characteristics and the study’s primary measures (reported and documented portal use) were calculated for descriptive analysis. Categorical variables were analyzed using frequency and percentage distributions, while continuous variables were examined using means and SDs.

Indices of reported and documented use were computed by averaging the medical/health and administrative items. Bivariate associations between reported use and documented use of the health portal were computed, as well as the associations of these with explanatory (independent) variables (demographics and DHL). Bivariate associations were also computed between all subindices (medical/health, administration, and overall) of reported and documented use.

For comparison between reported and documented use of services, both indices were aligned to qualitative ranges: none, low, and high. In the case of documented use, the transformation was none to none, ≥1 and ≤2 to low, and >2 to high. In reported use, the transformation was none to none, “less than once in this time period” and “1 to 2 times” to low, and “4 to 6 times” and “more than once in a given month” to high.

Lastly, we conducted linear hierarchical regressions to assess the association between documented and reported use and explanatory variables. Only variables that were significant in the bivariate analysis with either documented or reported use were entered. As years of computer use and using computers at work were highly correlated (*r*=0.50; *P*<.001), only the former was entered. The variables entered in the first step included the following: age, ethnicity, education, household size, self-rated health, and experience with computers. DHL was entered in the second step. We also reversed the order of the steps. Ethnicity was coded so that Jewish was 0 and Arab was 1. *R*^2^ and coefficients were reported.

## Results

### Participant Characteristics

Of 600 study participants, 314 (52.3%) were women and 286 (47.7%) were men, with a mean age of 70.45 (SD 7.54) years, ranging in age from 60 to 80 years. Over half (n=351, 58.5%) were Jewish and were married or cohabiting (n=378, 63%). Over half (n=365, 61%) completed more than a high school education, ranked themselves in the upper half of the social ladder (6 and above on a 10-point McArthur scale: n=449, 74.8%), and a quarter of the sample (n=150, 25%) found paying for medications difficult or very difficult. Nearly a third (n=179, 29.8%) rated their health as very good and good, 41.5% (n=249) as average, and nearly one-third (n=172, 28.6%) as bad or not so good. Most people lived either with another person in their household (n=258, 44%), or lived in larger households (n=198, 33%) and the rest, about a quarter (n=144, 24%), lived alone. Lastly, 65% (n=114) of the sample reported regularly using/used computers in their work, with an average of 18.35 (SD 15.53) years of experience using computers, and 58.3% (n=349) endorsed being very good at surfing the internet (see Table 1 for more details).

**Table 1.** Participant characteristics (N=600) and the association with documented and reported portal use.

Characteristics	Values	Association with documented portal use^a^	Association with reported portal use^a^
Gender, n (%)		*t*_598_=0.09^b^; *P*=.93	*t*_568_=0.61; *P*=.54
Women	314 (52.3)		
Men	286 (47.7)		
Age (y), mean (SD)	70.45 (7.54)	*r*=−0.08; *P*=.03^c^	*r*=0.05; *P*=.21
Ethnicity, n (%)		*F*_4,595_=7.13^d^, *P*<.001^c^	*F*_4,561_=6.45^d^; *P*<.001^c^
Jewish	351 (58.5)		
Muslim	164 (27.3)		
Christian	33 (5.7)		
Druze	13 (2.2)		
Other	38 (6.3)		
Marital status, n (%)		*F*_2,597_=1.32^d^;* P*=.27	*F*_2,563_=0.21^d^; *P*=.81
Single	29 (4.8)		
Married/cohabiting	378 (63)		
Divorced/widowed	193 (32.2)		
Education, n (%)		0.14, *P*<.001^c^	0.12, *P*=.003^c^
None, primary, or secondary	265 (44.2)		
Postsecondary	102 (17.0)		
BA degree	137 (22.8)		
MA degree and higher	96 (16.0)		
Paying for medications, n (%)		0.03; *P*=.54	–0.03; *P*=.51
Very easy	196 (32.7)		
Pretty easy	254 (42.3)		
Pretty difficult	88 (14.7)		
Very difficult	62 (10.3)		
Self-rated health		0.004; *P*=.92	−0.11; *P*=.01
Very bad	47 (7.8)		
Not so good	125 (20.8)		
Average	249 (41.5)		
Good	107 (17.8)		
Very good	72 (12)		
Household size		*r*=−0.08^d^, *P*=.04^c^	*r*=−0.03^d^, *P*=.57
1	144 (24)		
2	258 (44)		
3 and more	198 (33)		
Using/used computers at work, n (%)		*F*_1,172_=5.85^d^; *P*=.02^c^	*F*=2.99^d^; *P*=.09
Yes	114 (65)		
No	60 (34.5)		
Missing	426 (71.0)		
Years of using computers, mean (SD)	18.35 (15.53)	*r*=0.14; *P*<.001^c^	*r*=0.22; *P*<.001^c^

a Statistics reported are Pearson *r*, unless otherwise stated.

b Statistics is *t *test*.*

c Significant associations.

d Statistics is *F*; in the case of ethnicity and marital status, the 2 large groups were compared.

### Documented and Reported Portal Use

Documented and reported use are presented for each service (Multimedia Appendix 2) and for the averaged indices: medical/health, administrative, and overall.

The overall reported and documented indices were aligned for comparative purposes, so that both reported and documented use included 3 categories: none, low, and high. The frequency scale of the averaged documented use of the types of services on the portal (measured as the number of times, continuous) was transformed into categories: none, low (≥1 and ≤2), and high (>2). Categories on the scale of reported use were transformed into none, low (including “less than once in this time period” and “1‐2 times”), and high (the 2 upper response options). The documented and reported use of the portal is presented in Table 2.

**Table 2.** Reported and documented use of the HMO portal^a^.

Percentage of portal use by services	Reported^a^, n (%)	Documented, n (%)
None	46 (8.1)	372 (62.0)
Low	123 (21.7)	223 (37.2)
High	397 (70.1)	5 (0.8)

a There was missing data on the reported use for 34 respondents.

The data indicate that reported use is higher than documented use. The data from the HMO portal indicate that the majority of the participants (n=372, 62.0%) did not use the portal at all in the past year, whereas only 8.1% (n=46) reported not using it in the past 6 months. An opposite pattern was observed for use defined as low: documented use of the portal under this category was about a third (n=223, 37.2%), but only a fifth of participants reported using the portal at this use level (n=123, 21.7%). Most participants reported using the portal at high levels (n=397, 70.1%), whereas documented high use was reported for only a few participants (n=5, 0.8%). The data on specific types of services used (Multimedia Appendix 2) also consistently showed higher reported use than documented use.

The association between documented and reported use of the portal was positive and significant (*r*=0.22; *P*<.001), such that higher documented use was positively associated with higher reported use (significant associations are given in Table 3). This association was also found at the service-use level, both for medical/health-related services between documented and reported use (*r*=0.16; *P*<.001) and for administrative services between documented and reported use (*r*=0.19; *P*<.001), such that greater engagement in one activity was associated with greater use of the other activity. Table 3 also indicates a significant positive association between the 2 types of services in the portal, medical/health-related and administration-related, in both the reported (*r*=0.41; *P*<.001) and documented use (*r*=0.56; *P*<.001).

**Table 3.** Associations (Pearson *r* and 2-tailed *P* value) between use indices (reported and documented) and digital health literacy (DHL).

Associations	1	2	3	4	5	6	7	8
1. Reported medical/health services	—^a^							
2. Reported administrative services	0.41; *P*<.001	—						
3. Reported services index	0.88; *P*<.001	0.80; *P*<.001	—					
4. Documented medical/health services	0.16; *P*<.001	0.27; *P*<.001	0.24; *P*<.001	—				
5. Documented administrative services	0.10; *P*=.02	0.19; *P*<.001	0.16; *P*<.001	0.56; *P*<.001	—			
6. Documented services index	0.14; *P*<.001	0.25; *P*<.001	0.22; *P*<.001	0.83; *P*<.001	0.91; *P*<.001	—		
7. Digital health literacy	0.03; *P*=.54	0.20; *P*<.001	0.11; *P*=.01	0.17; *P*<.001	0.09; *P*=.04	0.17; *P*<.001	^—^	
8. Using digital resources	0.11; *P*=.02	–0.01^b^	0.06^b^	0.02^b^	0.05^b^	0.04^b^	0.04^b^	—

a Not applicable.

b All *P* values are >.05.

### Bivariate Associations With Portal Use

The association between documented portal use and background variables is displayed in Table 1 in the third column (with significant associations/differences mentioned in Table 1). Pearson coefficients were computed for continuous variables, and a 2-tailed *t* test or ANOVA was used for categorical variables. Age (*r*=−0.08; *P*=.03), higher education (*r*=0.14; *P*<.001), and ethnicity (being Jewish, *F*_4,595_=7.13; *P*<.001) were significantly associated with documented portal use, as well as household size (*r*=−0.08; *P*=.04) and using or having used computers at work (*F*_1,172_=5.85; *P*=.02). Documented portal use was not significantly associated with gender, marital status, being able to cover the expense of medications, and self-rated health (all *P* values >.05).

A somewhat similar pattern emerged between reported portal use and background variables (last column in Table 1): higher education (*r*=0.12; *P*=.003), ethnicity (being Jewish, *F*_4,561_=6.45; *P*<.001), and years of experience with computers (*r*=0.22; *P*<.001) were significantly associated with reported portal use; unlike documented use, self-rated health was associated with reported use, with participants reporting worse health reporting more portal use (*r*=−0.11; *P*=.01).

We also examined all portal use indices in relation to DHL (both the cognitive capacities and the use of digital devices; Table 3). Documented portal use indices (administrative and medical-related indices, and the overall index) were significantly associated with DHL, but not with the use of various digital devices (all *P* values >.05), except for the association between digital device use and medical-related services in the portal (*r*=0.11; *P*=.02).

### Multivariate Associations Explaining Portal Use (Reported and Documented)

We regressed background and DHL variables on reported and documented portal use across services. We included only variables that were significantly associated with combined services—either reported or documented—at the bivariate level. Only one variable, “using or having used computers at work,” was excluded from the analysis due to high shared variance with the related question of “experience with computers” (*r*=0.50; *P*<.001). Table 4 presents the results of the 2 regressions.

**Table 4.** Hierarchical linear regression on documented and reported portal use.

Characteristics	*B*	SE(*B*)	β	*P* value
Outcome: documented portal use^a^				
Age	0	0	−0.09	.04
Ethnicity	−0.17	0.04	−0.22	<.001
Education	0.01	0.01	0.05	.37
Household size	0.01	0.01	0.05	.36
Self-rated health	0	0.02	0.01	.86
Experience with computers	0	0	0.13	.02
DHL^b^	0.01	0.01	0.02	.97
Outcome: reported portal use^c^				
Age	0	0	0.04	.43
Ethnicity	−0.1	0.1	−0.06	.32
Education	0	0.03	0.01	.83
Household size	0	0.02	−0.01	.79
Self-rated health	-0.12	0.04	-.15	<.001
Experience with computers	0.01	0	.23	<.001
DHL	0	0.002	.04	.46

a *R*2 for the documented portal use outcome is 12.5%.

b DHL: digital health literacy.

c *R*2 for the reported portal use outcome is 7.1%.

As shown in Table 3, the documented use of the portal was explained better (adjusted *R*^2^=12.5%) than reported use (adjusted *R*^2^=7.1%), with documented use significantly explained by the ethnicity of patients (with higher use among patients who identified as Jewish than among those who identified as Arab) and reported experience with computers (the more experience, the more use), while reported use was significantly explained by experience with computers and self-rated health, with education, ethnicity, and DHL not significantly associated with reported use. Notably, DHL, while significantly associated at the bivariate level, did not reach significance at the multivariate level in both analyses, possibly due to shared variance with other explanatory variables (eg, *r*=0.46; *P*<.001, *r*=0.49; *P*<.001, and *r*=0.31; *P*<.001, for education, experience with computers, and self-rated health, respectively).

## Discussion

### Principal Results

This study focused on portal use—documented and reported—among a relatively vulnerable, growing in numbers, and highly relevant population segment—older adults. The design of the study—relying on both sources of information—allowed the researchers to identify and quantify a significant gap between patients’ perceptions of their activity and their documented behavior. The study found a significant discrepancy between patients’ self-reported use of services on their health portal and the documented use of these services by the HMO, supporting H1 that reported use will be higher than documented use. While only 8.1% (n=46) of participants reported not using the portal at all, the retrospective analysis of the CHS database indicated that 62% (n=372) did not use the services on the portal at all. This substantial gap underscores a potential overestimation of use of services when relying solely on self-reported data, suggesting that both indicators should be used in studies recording and explaining portal use.

Despite this disparity, a positive correlation was found between self-reported use and documented use of the portal (*r*=0.22; *P*<.001). This positive association suggests that although not compatible, each measure represents a different aspect of use of services. When examining factors associated with portal use in multivariate analyses, the hypotheses on age and gender (H2: negative association with age and more use among women) were not supported (though the age component of H2 was supported in a bivariate analysis). Key predictors of documented portal use included ethnicity (higher use among participants who identified as Jewish than among those who identified as Arab) and reported experience with computers (more experience correlated with greater use, addressing the RQ). In contrast, reported use was significantly explained by self-rated health (increased use among participants reporting worse health) and by reported experience with computers. Additionally, the model for documented use demonstrated better explanatory power (adjusted *R*^2^=12.5%) compared to reported use (adjusted *R*^2^=7.1%). DHL was associated with most use indices in a bivariate analysis, but was not significantly associated with either type of use in multivariate analyses, thus lending mixed support for H3. Lastly, both medical/health-related and administrative services within the portal were positively correlated in both reported (*r*=0.41; *P*<.001) and documented use (*r*=0.56; *P*<.001).

### Comparison With Prior Work and Interpretation

The substantial gap observed between self-reported and documented portal use in this study is a major finding, echoing concerns highlighted in previous literature regarding the accuracy of self-reported use of digital health services. Studies have consistently shown that self-reported data can often reflect an overestimation of portal use. For instance, Beal et al [8] indicated that measurements of patient portal use in research often vary widely, with inconsistent definitions and a reliance on simplified metrics, such as “at least one login,” which may not reflect meaningful engagement or use of services. Tavares and Oliveira [22] also noted that awareness and registration do not necessarily translate to frequent use, finding that only 30% of users regularly checked their electronic health records portal. The current study’s finding of 62% (n=372) documented nonuse compared with 8.1% (n=46) reported nonuse attests to the limitations of self-report and the need to augment self-reports with objective, tracked data to better understand patient engagement. The disparity between perceived and recorded measures is analogous to the low concordance between self-reported and externally measured DHL/eHealth literacy [23] as well as between self-reported and documented health care use for periods exceeding a month [23]. Another relevant comparison is the assessment of stress, which overwhelmingly encompasses both self-reported stress and physiological measures. These two are also only moderately correlated and are considered to capture different, distinct aspects of the stress response, each considered valid [24]. Other examples are smoking or physical activity, assessed by both self-reports and objective measures (accelerometers and the presence of cotinine [a metabolite of nicotine], respectively).

As there was no incentive for high reported use, interpretations can vary, ranging from measurement explanations, cognitive-motivational explanations, and broader identity signaling or symbolic engagement explanations. Measurement explanations address the possibilities that participants perceived surrogate/assisted use (clinic staff or family members) as their own use; that respondents may have experienced recall bias or estimation bias, so that they genuinely believe they use the portal more frequently than they actually do; or that an acquiescence bias occurred, whereby some respondents tended to answer “yes” to an interviewer. Conversely, database records may be incomplete because of the definition of a completed transaction or errors in linking activity to individual participants. Therefore, electronic records should not necessarily be regarded as an error-free gold standard, and thus the gap could be interpreted as measurement disagreement, as neither self-report nor database documentation necessarily captures precisely the same construct.

A more social-psychological interpretation pertains to constructs such as self-efficacy and self-perception. Self-efficacy theory could suggest that individuals with high digital health self-efficacy may report portal use partly based on confidence, perceived competence, or familiarity, rather than documented frequency [25]. Relatedly, Bem’s Self-Perception Theory proposes that people infer attitudes and identities from observations of themselves, constructing narratives about who they are. Specifically, respondents may infer that portal use is consistent with a self-image of caring about their health, being engaged with their health, and using modern technology. Under this interpretation, over-reporting is identity-consistent self-description [22]. The technology acceptance model could also explain the findings through the intention-behavior gap: respondents may have reported intended use, expected use, or favorable attitudes, instead of documented behavior. Valuing and intending to use the portal may have become using the portal [26]; respondents understood something other than what was intended.

The reported-documented use gap may be broadly interpreted through identity signaling theory or digital participation/symbolic technology use research. Identity signaling theory proposes that behaviors communicate desired identities to others and even to oneself [27]. Digital health technologies increasingly function as symbols of responsibility, proactivity, health consciousness, and technological competence. Thus, respondents may have reported portal use in a manner consistent with their identity as an engaged, responsible, and digitally connected person, even when objective use remains low. While identity signaling has not been studied in patient portals specifically, it was used to explain other digital health behaviors, such as health-app adoption and wearable use [28].

The perspective of digital participation views portal use as part of a broader digital inclusion and citizenship [29]. Under this perspective, the central question becomes: “Does an individual feel they participate in digital health care?” rather than “How many times have they logged in?” Hence, portal use may function as a marker of digital inclusion, and respondents who see themselves as digitally connected may report higher use because they either identify as digitally engaged, feel connected to modern health care, or find that portal membership itself becomes meaningful. Thus, reporting use reflects perceived participation in digital health care ecosystems rather than observed activity.

In conclusion, on the interpretations of the reported-documented use gap, rather than viewing self-reported portal use solely as a proxy for behavior, “use” may be more appropriately understood as a multidimensional construct that reflects both documented engagement and participants’ self-perceptions, identity signaling, and symbolic affiliation with digital health care, thereby explaining why subjective reports and objective usage metrics do not necessarily converge.

The identified demographic disparities in documented portal use align with, and also nuance, existing research. The finding that older age was associated with less documented portal use in the bivariate analysis is largely consistent with numerous studies reporting that older adults generally trail the general population in adopting digital health technologies. However, some literature presents mixed findings on age, with Carini et al [7] noting both increasing use with age and a lack of homogeneity in results, while Woods et al [10] found no significant differences in portal logins by age among already enrolled users. The current study, focusing on an older adult population (mean age 70.45 y), records the challenge faced by this demographic group in portal use.

Regarding ethnicity, our finding of higher documented use among Jewish participants compared with Arab participants supports the broader understanding that patient portal use is often lower among racial and ethnic minorities. Previous research suggests that White patients are generally more likely to use patient portals and that patients’ race and ethnic backgrounds are associated with perceptions of need for, and access to, portal services [2,6,30]. However, it is also important to note that some national studies [10,31], after adjusting for other factors, have not identified significant associations between patients’ race and ethnic backgrounds and patient portal access or use. The significant association between ethnicity and *documented* portal use observed in our multivariate model (as opposed to no significant association of ethnicity with *reported* use) may be due to different degrees of acquiescence bias in reporting among the groups [32,33] or to differences in actual use associated with reduced internet infrastructure and resources available in rural areas, inhabited more by Arabs.

The significant association of reported experience with computers for both self-reported and documented portal use is well-supported in prior studies, either reviews or empirical work. Limited digital literacy, computer skills, and internet access are frequently cited as significant barriers to patient portal adoption [7,10,13,14]. This consistency across studies on prior experience with computers underscores the fundamental importance of previously developed digital skills for effective portal engagement.

Our findings on DHL—association with reported and documented use only at the bivariate level and not in a multivariate analysis—align with the mixed results in the literature. For example, van der Vaart et al [13], who examined documented portal use, found it associated with eHealth literacy, as well as other works [34,35] which identified eHealth literacy as positively associated with *reported* portal use. However, Xie concluded in her review [36] that the evidence is inconclusive, as other studies point to no association [37,38]. Still, this result could stem from the shared variance with other related variables in the multivariate analysis.

The difference in explanatory power between models for documented vs reported use (*R*^2^=12.3% vs *R*^2^=5.3%) could reflect a measurement issue or a substantive difference. Measurement-wise, documented portal use is an objective measure with relatively low noise, whereas self-reported use is subjective and likely contains greater measurement error. These differences in measurement properties alone can influence *R*^2^ values, independent of any true discrepancy. Alongside, this result may indicate that objective measures capture a different angle of user behavior and its drivers. It should be noted that studies using the technology acceptance model and its extensions [22,34] explained higher variance than that found in our study, though they addressed reported usage and not documented usage. Still, most variation in portal engagement remains unexplained.

### Strengths and Limitations

This study demonstrates methodological strengths. A primary strength is its robust dual-data approach, which directly addresses a paucity in the literature by comparing both self-reported portal use and objective, documented use data retrieved directly from the HMO database. Furthermore, the study strategically focused on a vulnerable, growing, and highly relevant population segment—older adults—and used a medium-sized sample of 600 individuals aged 60 years and older. This demographic is critical to study because older adults often need to manage complex chronic conditions, have high health care needs, and stand to benefit significantly from portal functionalities, yet often face unique challenges with technology adoption. Finally, the study moves beyond basic demographics by incorporating specific digital variables, such as DHL and reported experience with computers, into its analysis.

Several limitations should be considered when interpreting these findings. First, the nonidentical temporal frames and scales used for documented (frequency during the year of 2024) and reported use (addressed past 6 months, ranging from “not at all” to “more than once a month”) should be considered. The latter time frame (6 months) was intended to reduce recall bias, yet it may have made direct comparisons difficult and introduced measurement bias. However, we did not compare exact quantities but rather qualitative ranges, and when comparing reported and documented use we focused on the category of no use, which was identical in both measures. Relatedly, though the time frame was constructed to be shorter to facilitate the accuracy of reported use (6 months rather than a year), some degree of recall error or forgetfulness may be attributed to age-related memory challenges among people aged 60 years and older. Second, while the study directly addressed the gap between self-reported and documented use, it relied on self-reported data for some variables, such as computer experience and DHL. Additionally, some participants might have had clinic staff or family members use the portal on their behalf (surrogate/assisted use), which could influence their self-perception of “use” without actual personal operation. Notably, this interpretation of surrogate use would emphasize the gap between reported and documented use even more. Third, the cross-sectional design of the survey limits the ability to establish causality or observe changes in portal use patterns and their explanatory variables over time. Longitudinal studies are essential for understanding the evolution from initial awareness to sustained adoption of patient portals. Fourth, the study was conducted within a single HMO in Israel, which—though serving more than half the country’s population—may limit the generalizability of findings to other health care systems or populations with different cultural, socioeconomic, or technological contexts. Fifth, the response rate of 20.4% may be considered relatively low. Though this response rate is not uncommon in a population sample with no paid incentive [39], it may have introduced selection bias, which in turn may have biased estimates. Furthermore, the study analyzed the population aged 60 years and older as one homogeneous sample with regard to age. Further studies could contribute more in-depth analyses of stratified age groups among the older population.

### Conclusions

This study reveals a substantial gap between patients’ perceptions of their health portal use and their documented use, with self-reported use significantly higher than documented use. This highlights a critical challenge for health care systems and reinforces the necessity of also using objective metrics to comprehensively assess patient engagement. The identification of background variables associated with documented portal use underscores the persistent digital divide, which affects vulnerable populations, including older adults and certain ethnic minority groups.

Bridging this divide and enhancing patient engagement calls for integrating interventions in health care that are culturally sensitive, tailored to specific demographic needs and digital experience levels, including DHL. By addressing these factors, health care systems can move toward more equitable and effective patient portal use, ultimately leveraging these tools to improve health outcomes for all.

## Supplementary material

10.2196/93057 Multimedia Appendix 1 Use of portal (reported and documented).

10.2196/93057 Multimedia Appendix 2 Frequency of portal-documented services (N=600).

## Abbreviations

CHS: Clalit Health Services
DHL: digital health literacy
HMO: health maintenance organization
RQ: research question
