# Supplementary material for: Gaps Between Self-Reported and Documented Use of a Health Care Service Portal Among Older Adults: Cross-Sectional Survey and Retrospective Electronic Data Study
Source: JMIR Aging. 2026 Oct 13;9:e93057. doi: 10.2196/93057 (PMC13661037; doi:10.2196/93057)
Supplement: Supplementary file 1 [file aging-v9-e93057-s001.docx]

**Multimedia Appendix 1**.

Use of portal (reported and documented)

Digital Health Literacy (DHL)

When you search online for information on health, how easy or difficult is it for you …

|  | Very easy | Easy | Difficult | Very difficult | Irrelevant/DK/refusal (only spontaneously) |
| --- | --- | --- | --- | --- | --- |
| … to use the proper words or search query to find the information you are looking for? |  |  |  |  |  |
| … to find the exact information you are searching for ? |  |  |  |  |  |
| … to understand the information? |  |  |  |  |  |
| … to judge whether the information is reliable? |  |  |  |  |  |
| … to judge whether the information is offered with commercial interests? |  |  |  |  |  |
| … to visit different websites to check whether they provide similar information about a topic? |  |  |  |  |  |
| … to judge whether the information is applicable to you? |  |  |  |  |  |
| … to use the information to help solve a health problem? |  |  |  |  |  |

HLS19-DIGI-DD (Digital Devices)

In a typical week, how many days do you use the following digital resources for getting health related information?

|  | Less than once per week | 1-3 days per week | 4-6 days per week | Once a day | Irrelevant/DK/refusal (only spontaneously) |
| --- | --- | --- | --- | --- | --- |
| Websites |  |  |  |  |  |
| Social Media *(incl. online forums)* |  |  |  |  |  |
| A digital device related to health or health care *[Interviewer instruction e.g. pedometer, smart watch, fitness bracelet, etc.).* |  |  |  |  |  |
| Health app on your mobile phone  *[Interviewer instruction e.g. to calculate calorie consumption, support medication intake, measure physical activity, promote healthy sleep, etc.]* |  |  |  |  |  |
| Digital interaction with your health system *[Interviewer instruction e.g. online appointments, access to personal health records electronic delivery/transmission of medical tests, digital communication with a provider, etc.]* |  |  |  |  |  |
| Other |  |  |  |  |  |

Reported Portal use

We previously asked you a general question about your interactions with the healthcare system.

We would now like to ask more specifically about actions you may have undertaken in the last six months.

How often have you used each of the following services of the computerized system of your HMO in the past six months?

|  | Not at all | less than once in this period | 1-2 times | 4-6 times | more than once in a month | DK/refuses/irrelevant (interviewer: not to suggest; only if spontaneous) |
| --- | --- | --- | --- | --- | --- | --- |
| Clinical Summaries, authorizations/certificates (e.g., sick leave, summary of medical history) |  |  |  |  |  |  |
| Referrals for tests/procedures |  |  |  |  |  |  |
| Requests from physician (such as prescriptions) |  |  |  |  |  |  |
| Consultation w. administrators |  |  |  |  |  |  |
| Approval for funding services outside CHS system |  |  |  |  |  |  |
| Payment refunds |  |  |  |  |  |  |
