# Supplementary material for: Gaps Between Self-Reported and Documented Use of a Health Care Service Portal Among Older Adults: Cross-Sectional Survey and Retrospective Electronic Data Study
Source: JMIR Aging. 2026 Oct 13;9:e93057. doi: 10.2196/93057 (PMC13661037; doi:10.2196/93057)
Supplement: Supplementary file 2 [file aging-v9-e93057-s002.docx]

**Multimedia Appendix 2**.

**Frequency of portal documented services (N=600) (N, %)**

**Activity	None	Low	high**

**1-2 times	>2 times**

Clinical Summaries,

authorizations/certificates	558 (93.0)	27 (4.5)	15 (2.5)

Referrals for tests/procedures	526 (87.7)	40 (6.7)	34 (5.6)

Requests from physcian	433 (72.2)	54 (9.0)	113 (18.8)

Consultation with administrators	550 (91.7)	26 (4.3)	24 (4.0)

Approval for funding services

outside CHS system	573 (95.5)	18 (3.0)	9 (1.5)

Payment refunds	597 (99.5)	3 (0.5)	--

**Frequency of portal self-reported services (N, %)**

**Activity	None	low	high	refuses/	<=2	(>2)	irrelevant**

Clinical Summaries,

authorizations/certificates	184 (30.7)	210 (35.0)	148 (24.7)	58 (9.7)

Referrals for procedures	108 (18.0)	253 (42.2)	188 (31.3)	51 (8.5)

Requests for prescriptions	122 (20.3)	232 (38.7)	190 (31.7)	56 (9.3)

Consultation w. administrators	409 (68.2)	89 (14.8)	24 (4.0)	78 (13.0)

Authorization for procedures

outside CHS system	371	(61.8)	110 (18.3)	51 (8.5)	68 (11.3)

Payment refunds	264 (44.0)	207 (34.5)	70 (11.7)	50 (9.8)
